# Differentially Expressed microRNAs in Peritoneal Dialysis Effluent-Derived Exosomes from the Patients with Ultrafiltration Failure

**DOI:** 10.1155/2022/2276175

**Published:** 2022-08-31

**Authors:** Weifei Wu, Xu Wu, Zhiqun Cheng, Zhenzhen Yang, Minhui Lu, Jing Cheng

**Affiliations:** Department of Nephrology, Huzhou Central Hospital, Affiliated Central Hospital Huzhou University, Huzhou 313000, Zhejiang, China

## Abstract

**Background:**

Ultrafiltration failure remains one of the most severe complications of long-term peritoneal dialysis (PD), which results in death. This study aimed to characterize the circulating exosomal microRNA (miRNA) profiles associated with ultrafiltration failure and explore its underlying mechanisms.

**Methods:**

Exosomes were isolated from the peritoneal dialysis effluent (PDE) of patients with ultrafiltration failure or success using the ultracentrifugation method, and then transmission electron microscopy (TEM), nanoparticle tracking analysis (NTA), and western blot were used for exosome characterization. After that, the isolated exosomes were sent for small RNA sequencing, and eight differentially expressed miRNAs (DE-miRNAs) were chosen for RT-qPCR validation.

**Results:**

TEM, NTA, and western blot revealed that exosomes were successfully isolated. After sequencing, 70 DE-miRNAs involved in ultrafiltration were identified, including 41 upregulated ones and 29 downregulated ones. Functional analyses revealed that these DE-miRNAs were significantly enriched in pathways of cancer, ubiquitin-mediated proteolysis, axon orientation, and the Rap1 and Ras signaling pathways. In addition, the consistency rate of RT-qPCR and sequencing results was 75%, which indicated the relatively high reliability of the sequencing data.

**Conclusions:**

Our findings implied that these DE-miRNAs may be potential biomarkers of ultrafiltration failure, which would help us to discover novel therapeutic targets/pathways for ultrafiltration failure in patients with end-stage renal disease.

## 1. Introduction

Chronic kidney disease (CKD), which can result in the gradual loss of kidney function, is the leading cause of end-stage renal disease (ESRD). Peritoneal dialysis (PD) is one of the major forms of renal replacement therapy for patients with ESRD. Currently, CKD affects approximately 700 million people worldwide, and individuals with late stages of CKD are at a high risk of developing kidney function failure, requiring dialysis or transplantation [1]. In many countries, increasing evidence have demonstrated that the outcomes of patients undergoing PD are comparable to or better than those undergoing hemodialysis [2]. PD is generally more convenient, more cost effective, and preserves the residual renal function better than hemodialysis [3]. However, continuous exposure to glucose in the peritoneal dialysate inevitably induces injury that negatively affects peritoneum function, resulting in ultrafiltration failure and PD discontinuation [4].

Peritoneal fibrosis is the main pathological condition of ultrafiltration failure and is regulated by processes of inflammation, angiogenesis, and epithelial–mesenchymal transition (EMT) [5]. TGF*β*1-meditated EMT has been confirmed as the central regulator of organ fibrosis. Most PD solutions contain glucose. Glucose degradation products activate TGF*β*1 signaling and downstream profibrotic molecules such as the smad family and snail [6,7]. TGF*β*1 increases VEGF-A production in mesothelial cells and fibroblasts, which promote the EMT process and fibroblast proliferation, leading to the expansion of peritoneal fibrosis [8]. Currently, there are no specific biomarkers or well-established therapies for peritoneal fibrosis. Peritoneal functional assessment mainly relies on the peritoneal equilibration test (PET). Considering that ultrafiltration failure occurs only in the advanced stages of peritoneal fibrosis, PET data in clinical practice might be available too late to monitor peritoneal function. Although invasive peritoneal biopsy could be used for the pathological diagnosis of PD-related peritoneal injury, the results do not reflect global peritoneal structural changes [9,10]. Thus, it is highly desirable to establish suitable biomarkers and therapeutic targets for disease progress prediction and to allow patients to improve their survival during long-term PD.

Exosomes serve as key mediators in intercellular communication by transferring diverse components, including DNA, microRNA (miRNA), proteins, and lipids [11]. Cytokine stimulation induces the release of abundant exosomes from original cells and affects peritoneal membrane function, thereby contributing to the development of fibrosis [12]. Recent studies have demonstrated the importance of exosomes in peritoneal dialysis effluent (PDE) samples, and they might be used as the ideal source of biomarkers for PD patients owing to their noninvasiveness and convenience in collection [13–15]. Moreover, miRNAs have attracted attention as reliable biomarkers and potential targets for precise therapies. miRNAs are small noncoding RNAs (21–25 nt) that function as major post-transcriptional regulators through interactions with mRNAs and induce their destabilization and translation [16]. Many miRNAs display aberrant expressions in dialysate effluents or serum and are implicated in the activation of profibrotic signaling in PD patients [17]. For instance, miR-302c is observed to be downregulated in peritoneal mesenchymal cells (PMCs) isolated from PDE, and it modulates PD-related fibrosis via the modulation of connective tissue growth factors [18]. miR-21 and miR-589 levels are also dysregulated in PDE, which are correlated with changes in peritoneal transport characteristics [19]. However, few studies report the specific miRNA expression status in PDE exosomes of patients with ultrafiltration failure. A recent study revealed that the miRNAs enclosed in exosomes were more stable than circulating miRNAs as the exosomal membrane structure acts as a barrier against enzyme degradation [20]. This observation provides a basis that exosomal miRNAs might be ideal biomarkers for the diagnosis of certain diseases.

Therefore, in this study, we aimed to investigate the underlying miRNA-related mechanisms in peritoneal ultrafiltration failure. Exosomes were isolated from the PDE samples of patients with ultrafiltration failure and success and then were submitted for small RNA sequencing. Differentially expressed miRNAs (miRNAs) were screened with the thresholds of |log_2_fold change (FC)| > 1 and *P* < 0.05, and functional analyses were performed. After that, eight DE-miRNAs, namely, hsa-miR-1273c-p3, hsa-miR-125a-5p, hsa-miR-1277-5p, hsa-miR-132-3p, hsa-miR-296-3p, hsa-miR-25-5p, hsa-miR-155-5p, and hsa-miR-708-5p, were selected for real-time quantitative PCR (RT-qPCR) verification. Our work will improve our understanding of ultrafiltration failure and provide potential targets for the treatment of ultrafiltration failure in patients with end-stage of renal disease.

## 2. Materials and Methods

### 2.1. Regents and Antibodies

Primary antibodies utilized in our study: anti-HSP70 antibody (1 : 1000, Proteintech, Chicago, USA), anti-CD63 antibody (1 : 1000, ABclonal, Boston, USA), anti-CD81 antibody (1 : 1000, Abcam, MA, USA); secondary antibodies include HRP-labeled goat antirabbit IgG (1 : 1000, Beyotime, Shanghai, China). A chemiluminescence kit was purchased from Millipore (Darmstadt, Germany).

### 2.2. Patients and PDE Collection

Patients were derived from the PD center of Huzhou Central Hospital. Three patients experienced ultrafiltration failure during stable PD treatment and were diagnosed with type I peritoneal ultrafiltration failure according to the PET assay. Meanwhile, three subjects with similar demographics and normal peritoneal function were recruited. Informed consent was obtained from all subjects. This study was approved by the Institute of Research Ethics Review Committee in Huzhou Central Hospital (No. SH9H⁃2020⁃T23⁃1).

Approximately 500 mL of overnight PDE (abdominal retention >8 h) was collected from all subjects and was immediately processed for exosome separation. General clinical information of patients was recorded, including PDE routine testing, assessment of the residual renal function, peritoneal equilibrium test parameters, residual urine volume, ratio of dialysate creatinine to serum creatinine at 4 h (4 h D/Pcr), a sodium sieving ratio at 1 h (1 h D/Pna), material transport area coefficient of creatinine (MTAC), and ultrafiltration volume (UF) (Table 1). All the patients diagnosed with primary nephropathy were in accordance with the diagnosis of ESRD and received regular PD replacement therapy for more than 3 months. The exclusion criteria were as follows: incomplete data; historical peritonitis at the time of enrollment or 3 months prior to enrollment; abdominal bleeding or other basic diseases such as neoplastic diseases, liver cirrhosis, serious heart, and lung disease; secondary nephropathy such as diabetic nephropathy, lupus nephritis, ANCA-associated vasculitis, and systemic amyloidosis; and the patients who were taking hormones and immunosuppressants were also excluded in this study.

The diagnosis of type I peritoneal ultrafiltration failure was based on the definition of the International Society for Peritoneal Dialysis in the year 2000 [21,22]. The enrolled patients underwent a modified method of PET with 4.25% dextrose fluid. All subjects underwent 4 h of dialysis exchange using 2 L of dialysis solutions at 3.86% glucose. Patients with ultrafiltration capacities of <400 mL and dialysate/serum creatinine concentration ratios (D/Pcr) of >0.81 could be diagnosed with ultrafiltration failure. The possible causes of ultrafiltration failure should be excluded to affect the judgment of the results, such as PD-associated peritonitis, improper adjustment of diet and dialysis management, hyperglycemia, posterior peritoneal leakage, and mechanical failure of the dialysis catheter (drift tube, leakage, etc.). Prior to PET experiments, we pre-exchanged overnight stays of 8–12 h.

### 2.3. Exosome Isolation and Characterization

The isolation of exosomes by ultracentrifugation was accomplished as previously described [23]. Briefly, the PDE samples were centrifuged at 300*g* for 10 min. The supernatant was transferred to a new tube and centrifuged at 3,000*g* for 15 min, followed by centrifugation at 12,000*g* for 30 min to remove cell fragments. After filtration with a 0.22 *μ*m filter, the supernatant was collected and centrifuged at 12,000*g* for 60 min to obtain the sediments of the membrane pellets. The pellets were resuspended with PBS and centrifuged at 12,000*g* for 70 min. The supernatant was then carefully removed, and the reserved precipitates were resuspended with 200 *μ*L of PBS, that was exosomes.

The characterization of the isolated exosomes was performed according to the ISEV suggestions in 2014 [24]. The concentrations of the isolated exosomes were detected using a BCA protein assay kit (Boster, Wuhan, China). Thereafter, a transmission electron microscope (TEM) was used to visualize the morphology of the exosomes, and nanoparticle tracking analysis (NTA) was performed to evaluate the size and distribution of the exosomes [25,26]. Finally, the expressions of HSP70, CD63, and CD81, which are the specific markers of exosomes, were detected by western blot with their corresponding antibodies [27].

### 2.4. Small RNA Sequencing and Bioinformatic Analysis

The isolated exosomes were then sent to Yanzai Biotechnology (Shanghai) Co. Ltd (Shanghai, China) for small RNA sequencing. Firstly, total RNA was extracted from the exosomes using RNAiso Plus reagent (TAKARA, Shiga, Japan), following the manufacturer's instructions. For each 250 *μ*L of exosome sample, 750 *μ*l of Trizol reagent was added for lysis, followed by 200 *μ*L of chloroform separation liquid. Then, isopropyl alcohol was added to the upper liquid to precipitate RNA from the exosomal suspension. The precipitates were resuspended with 75% ethanol and centrifuged at 12,000*g* at 4°C for 5 min. Finally, the precipitates were dissolved in 20 *μ*l of DEPC, and the purity and concentration of the extracted RNA were assessed by calculating the 260/280 optical density ratio using a microplate reader. After that, the extracted RNA was used for small RNA sequencing.

TruSeq small RNA sample prep kits (Illumina, San Diego, USA) were used for the construction of RNA libraries. Data preprocessing was performed using the ACGT101-miR software (LC Sciences, Texas, USA) [28]. Subsequently, miRNAs in the exosomes from PDE were annotated according to the miRbase database (https://www.mirbase.org/). Based on the criteria of |log_2_FC| > 1 and *P* < 0.05, DE-miRNAs were screened between the exosomes from the failed ultrafiltration PDE and successful PDE. Then, global analysis and hierarchical clustering analyses were performed for these DE-miRNAs. Afterward, the target genes of these screened DE-miRNAs were predicted using the target scan and miRDB software programs. Functional analyses of these DE-miRNAs, including Gene Ontology (GO) terms and Kyoto Encyclopedia of Genes and Genomes (KEGG) pathways, were carried out based on the Database for Annotation, Visualization, and Integrated Discovery (DAVID) [29]. The threshold of the significantly enriched GO terms and KEGG pathways was *P* < 0.05. The raw data of small RNA sequencing were uploaded to the Gene Expression Omnibus database, and the ID was GSE142819.

### 2.5. RT-qPCR Analysis

As for the identified DE-miRNAs, eight DE-miRNAs (four upregulated: hsa-miR-1273c-p3, hsa-miR-125a-5p, hsa-miR-132-3p, and hsa-miR-1277-5p; four downregulated: hsa-miR-155-5p,hsa-miR-708-5p, hsa-miR-25-5p, and hsa-miR-296-3p) were selected for RT-qPCR validation. The total RNA was extracted from the failed ultrafiltration PDE-associated exosomes (*n* = 3) and the successful ultrafiltration PDE-associated exosomes (*n* = 3) using RNAiso Plus reagent (TAKARA, Shiga, Japan) based on the manufacturer's instructions. The levels of the miRNAs were measured using the stem-loop method, and cel-miR-39 was served as a reference gene. The isolated total RNA was used to generate cDNA by PrimeScript™ II 1st Strand cDNA synthesis Kit TAKARA Biomedical Technology Co., Ltd., Beijing, China), according to the manufacturer's instructions. The sequences of all primers are listed in Table 2, and the primers were designed and synthesized by Sangon Biotech (Shanghai) Co., Ltd (Shanghai, China). RT-qPCR analysis was conducted on a PCR system (Applied Biosystem, USA) using Power SYBR Green Master kit (Thermo, USA). The RT-qPCR reaction was initiated at 50°C for 2 min, 95°C for 2 min, followed by a total of 40 cycles at 95°C for 15 s and 60°C for 60 s. The relative levels of hsa-miR-1273c-p3, hsa-miR-125a-5p, hsa-miR-155-5p, and hsa-miR-708-5p were calculated using the 2^−ΔΔCT^ method [30]. Each sample was analyzed in triplicate.

### 2.6. Statistical Analysis

All the data in this study were expressed as mean ± standard deviation. Graphpad Prism 6 (San Diego, CA, USA) and SPSS 22.0 (IBM, Armonk, NY, USA) were utilized for statistical analysis. Student's *t*-test was used for comparative analysis between the two groups. *P* < 0.05 was considered as the threshold for statistical significance.

## 3. Results

### 3.1. Characterization of the Isolated Exosomes

**Figure 1:**
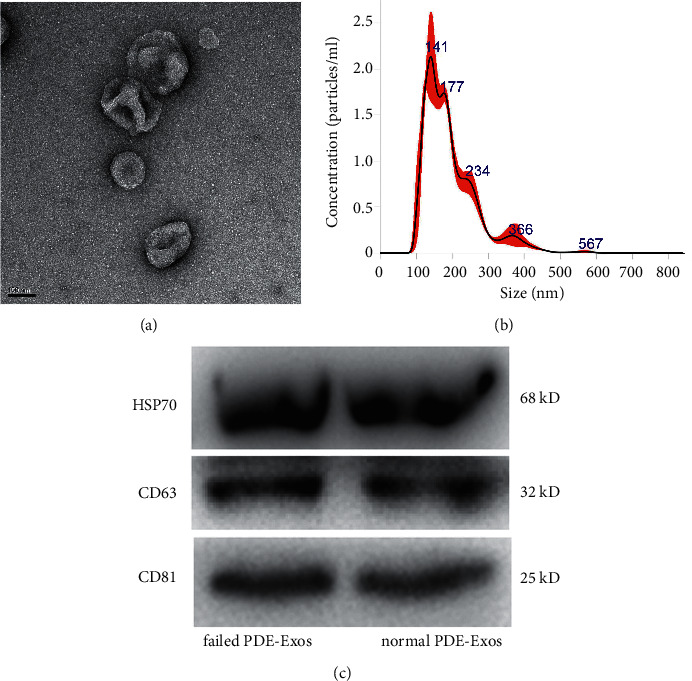
Characterization of the exosomes isolated from the peritoneal dialysis effluent (PDE). (a) Morphology of the exosomes observed by a transmission electron microscopy. Scale bar = 100 nm. (b) The particle size distribution of exosomes measured by nanoparticle tracking analysis. (c) Exosomes surface markers (HSP70, CD63, and CD81) detected using western blot.

Exosomes were isolated from the PDE samples and then characterized by TEM, NTA, and western blot. TEM results showed that the exosomes isolated from the PDE samples exhibited a cup-shaped or round morphology with a diameter of approximately 100 nm (Figure 1(a)). Then, NTA analysis revealed that the major peak of the isolated substance was approximately 141 nm (Figure 1(b)). Besides, western blot revealed that the exosome-specific markers HSP70, CD63, and CD81 were expressed in the isolated exosomes (Figure 1(c)). These results indicated that exosomes were successfully isolated from the PDE samples using the ultracentrifugation method.

### 3.2. Identification of DE-miRNAs between the Exosomes from the Failed Ultrafiltration PDE and Successful PDE

**Figure 2:**
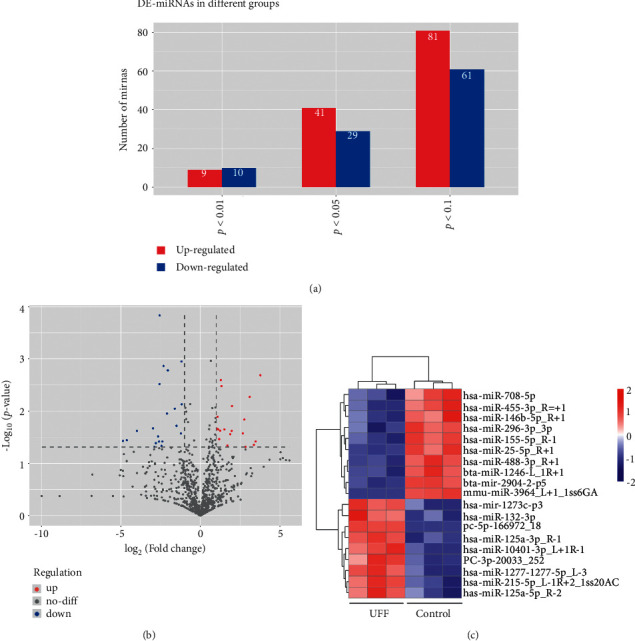
Screen of differentially expressed miRNAs (DE-miRNAs) between the exosomes from the failed ultrafiltration PDE and successful ultrafiltration PDE. (a) Based on the different *P* values *P* < 0.01, *P* < 0.05, and *P* < 0.1), DE-miRNAs were screened. (b) Volcano plots of the 70 DE-miRNAs with |log2 (fold change) |>1 and *P* < 0.05. The blue points represent the downregulation; the gray points represent the normal; and the red points represent the upregulation in the exosomes from the failed ultrafiltration PDE compared with the exosomes from the successful ultrafiltration PDE. (c) The bidirectional hierarchical clustering heatmap of the 19 DE-miRNAs with *P* < 0.01. UFF: the exosomes from the failed ultrafiltration PDE; control: the exosomes from the successful ultrafiltration PDE.

After small RNA sequencing, a total of 686 miRNAs were annotated in the exosomes from all PDE samples. According to the criteria of |log_2_FC| > 1 and *P* < 0.05, 70 DE-miRNAs were identified between the exosomes from the failed ultrafiltration PDE and successful PDE, including 41 upregulated miRNAs and 29 downregulated miRNAs (Figures 2(a) and 2(b)). Based on the threshold of *P* < 0.01, 19 DE-miRNAs were found between the two groups, including 10 downregulated miRNAs and 9 upregulated miRNAs (Figure 2(a)). The hierarchical clustering of these 19 DE-miRNAs is shown in Figure 2(c), which implied that these DE-miRNAs could properly distinguish the failed ultrafiltration PDE-derived exosomes from the successful ultrafiltration PDE-derived exosomes.

### 3.3. GO Terms and KEGG Pathway Analyses

**Figure 3:**
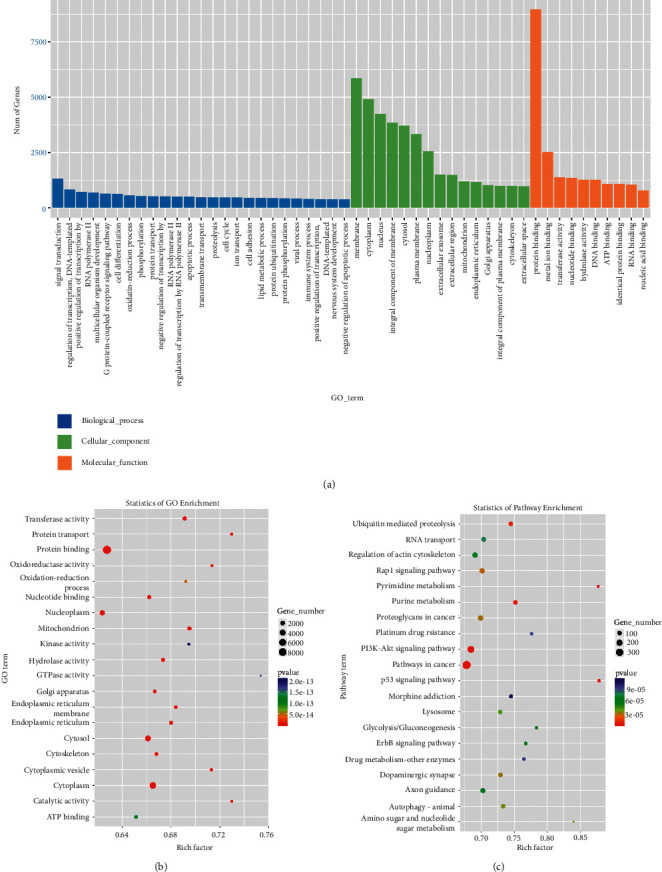
Functional analyses of the potential target genes of the identified DE-miRNAs. (a) The Gene Ontology (GO) terms analysis, including biological process, molecular function, and cellular component, performed based on DAVID. (b) The top 20 GO terms of the identified DE-miRNAs. (c) The top 20 Kyoto Encyclopedia of Genes and Genomes (KEGG) pathways of the identified DE-miRNAs. The size of the circles represents the number of genes. The color of the circle represents the *P*-value.

After that, these DE-miRNAs were submitted to predict the target genes using the target scan and miRDB databases, and 13176 target genes of DE-miRNAs were predicted. Then, these genes were used for functional analyses. Functional analyses revealed that these genes were mainly enriched in various GO terms and KEGG pathways. Figures 3(a) and 3(b) show the most enriched GO terms in biological process, molecular function (MF), and cellular component (CC), such as “protein binding,” “nucleotide binding,” “cytoplasm,” “cytosol,” “transferase activity,” “oxidation-reduction process,” and “ATP binding.” Additionally, the KEGG pathway enrichment analysis indicated that these genes were also significantly enriched in “ubiquitin-mediated proteolysis,” “Rap1 signaling pathway,” “pathways in cancer,” “PI3K-Akt signaling pathway,” “p53 signaling pathway,” “axon guidance,” and the “Ras signaling pathway.”

### 3.4. Validation of RT-qPCR

**Figure 4:**
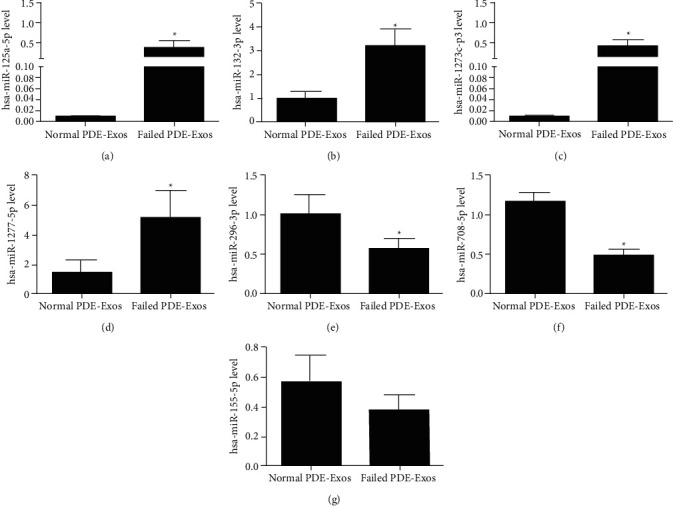
Verification of eight DE-miRNAs (four downregulated and four upregulated, one DE-miRNA failed to be detected due to its low abundance) in the exosomes from the failed ultrafiltration PDE and successful ultrafiltration PDE by RT-qPCR analysis. The levels of miR-125a-5p (a), miR-132-3p (b), miR-1273c-p-3 (c), miR-1277-5p (d), miR-296-3p (e), miR-708-5p (f), and miR-155-5p (g). ^*∗*^: *P* < 0.05, compared with the normal PDE-Exos group.

Further to verify the reliability of small RNA sequencing, four upregulated DE-miRNAs (hsa-miR-1273c-p-3, hsa-miR-125a-5p, hsa-miR-132-3p, and hsa-miR-1277-5p) and four downregulated miRNAs (hsa-miR-155-5p, has-miR-708-5p, hsa-miR-25-5p, and hsa-miR-296-3p) were chosen for validation. It is obvious that compared with the exosomes from the successful ultrafiltration PDE, the levels of hsa-125a-5p, hsa-132-3p, hsa-miR-1273c-p-3, and hsa-miR-1277-5p in the exosomes from the failed ultrafiltration PDE were all increased significantly (*P* < 0.05, Figures 4(a)–4(d)), whereas the levels of hsa-296-3p and hsa-miR-708-5p were both evidently decreased (*P* < 0.05, Figures 4(e) and 4(f)), which were in line with the expression patterns of sequencing results. However, there was no significant difference in the level of hsa-miR-155-5p between the failed ultrafiltration PDE-derived exosomes and successful ultrafiltration PDE-derived exosomes (*P* > 0.05, Figure 4(g)). For hsa-miR-25-5p, due to its low abundance in the exosomes from the successful and failed ultrafiltration PDE samples, it had not been detected. All these results indicated that the consistency rate of sequencing data and RT-qPCR results was 75% (6/8), indicating a relatively high reliability of the sequencing.

## 4. Discussion

Peritoneal ultrafiltration failure is associated with long-term PD and repeated peritoneal inflammation, which is the primary cause of PD withdrawal and is closely related to the poor prognosis of patients. MiRNAs, which serve as important post-transcriptional regulators, play important roles in tissue fibrosis and fibrosis-related diseases [31]. In our study, exosomes were isolated from the PDE of failed ultrafiltration patients and successful ultrafiltration patients and sent for small RNA sequencing. After sequencing, 70 DE-miRNAs were identified, including 41 upregulated miRNAs and 29 downregulated miRNAs. Functional analyses revealed that these DE-miRNAs were significantly enriched in pathways of cancer, ubiquitin-mediated proteolysis, axon orientation, the Rap1 signaling pathway, and the Ras signaling pathway. Finally, RT-qPCR showed that miR-1273c-p-3, miR-1277-5p, miR-132-3p, and miR-125a-5p were upregulated, while miR-296-3p and miR-708-5p were downregulated in the exosomes from the failed ultrafiltration PDE. These results would provide a basis for the potential mechanisms of exosomes associated with miRNA-mediated regulation of ultrafiltration failure in PD.

Accumulating studies have shown that patients undergoing PD begin to develop peritoneal structure changes, such as fibrosis and angiogenesis. Previous studies have reported that miRNAs displayed significant effects on peritoneal fibrosis both *in vitro* PMC assays and rodent models [32]. This study found that miR-1273c-p-3 miR-1277-5p, miR-132-3p, and miR-125a-5p were upregulated, while miR-708-5p and miR-296-3p were downregulated in the exosomes from the failed ultrafiltration PDE. The miR-125a gene is located at 19q13, and includes miR-125a-5p and miR-125a-3p. It has been reported that miR-125a-5p mainly functions as a tumor suppressor in several human cancers by downregulating target gene expression [33,34]. The decreased level of miR-125a in tumor tissues was correlated with aggressive pathologic features, and the mechanisms were mainly involved in regulating of EMT process. Studies have shown that miR-125a-5p could regulate tumor invasion, migration, and EMT by directly targeting the TAZ/EGFR pathway in both colorectal and ovarian cancers [35,36]. Besides, miR-125a-5p could regulate EMT-mediated migration and invasion by targeting the STAT3 signaling pathway in ESCC [37]. Additionally, miR-125a has also been reported to play an important role in regulating angiogenesis. In gastric cancer, miR-125a was capable of modulating the expression of VEGF-A, which is the main regulator of angiogenesis [38]. In hepatocellular carcinoma tissues, miR-125a was expressed ectopically and negatively correlated with MMP11 and VEGF-A expression [39]. The overexpression of miRNA-125a-5p could significantly inhibit the tumor growth and angiogenesis by repressing the VEGF-A secretion [40]. It has been reported that miR-125a-5p could serve as a diagnostic or prognostic biomarker for renal cell carcinoma [41], and exosomal miR-125a-5p has been shown to be higher in the placental tissues of women with preeclampsia and to suppress VEGF-A expression and angiogenesis in the peripheral blood of patients with preeclampsia [42]. EMT and angiogenesis were both major processes associated with the development of organ fibrosis and cancer; combined with these results, we speculate that the upregulation of miR-125a-5p may be involved in the enhancement of EMT and VEGF-A-mediated angiogenesis process, thus promoting peritoneal fibrosis.

Similarly, current studies on miR-708-5p and miR-296-3p were also mostly focused on different cancer types. Zhao et al. found that miR-708-5p could promote the EMT process by targeting the ZNF549 gene and activating the PI3K/AKT signaling pathway in the adenocarcinoma cells of the colon [43]. Another study on gliomas demonstrated that the silencing of miR-708 promoted cell growth and EMT transition by activating the SPHK2/AKT/*β*-catenin pathway [44]. Tian et al. [45] reported that miR-296-3p could inhibit the Wnt/*β*-catenin pathway by targeting SOX4 and exert antitumor effects in triple-negative breast cancer. Wang et al. demonstrated that miR-296-3p, acted as a tumor suppressor, could inhibit the migration and invasion of nonsmall-cell lung cancer cells by targeting APEX1 and regulating the PI3K/AKT/mTOR signaling pathway {[34] ^#^1145}. These indicated that miR-708-5p and miR-296-3p may be closely related to the EMT process and the development of diseases. Furthermore, miR-708 can impact the immunoreaction in the progression of diseases. It was found that the miR-708-5p level was increased in bacteria-infected human macrophages, and miR-708-5p mimics could reduce inflammatory cytokines secretion by targeting TRL4 [46]. Besides, several miRNAs, including miR-708-5p, could exert anti-inflammatory effects on endothelial cells in injured arteries by inactivating the NF-*κ*B pathway [47]. MiR-708 was identified as a negative regulator of TNF*α* and IL-1*β* molecules, and exacerbated TNF*α* and IL-1*β* signaling was involved in diverse inflammatory diseases [48]. Taken together, it can be inferred that miR-708-5p and miR-296-3p may participate in the regulation of inflammation and the EMT process in peritoneal fibrosis. According to a literature search, our study is the first to report the associated between exosomal miR-1273c-p-3/miR-1277-5p/miR-132-3p and ultrafiltration failure. The specific roles of miR-125a-5p, miR-1273c-pc-3, miR-1277-5p, miR-132-3p, miR-296-3p, and miR-708-5p in peritoneal fibrosis require further investigations.

In addition, these DE-miRNAs were significantly enriched in several major pathways, including ubiquitin-mediated proteolysis, axon orientation, the Rap1 signaling pathway, and the Ras signaling pathway. The ubiquitin–proteasome system (UPS) was a major regulatory mechanism of intracellular protein degradation, and controlled diverse cellular functions. UPS dysfunction can contribute to the development of cancer, autoimmune diseases, and organ fibrosis [49,50]. In PD mice models, the upregulation of nestin proteins could stimulate peritoneal fibrosis by protecting HIF1-*α* from proteasomal degradation [51]. UPS participates in the pathogenesis of organ fibrosis mainly by regulating the TGF*β*/Smad and Wnt/*β*-cantenin pathways [52,53], and the increased TGF*β* level could induce EMT and activate the STAT3 signaling in human PMCs [54]. Interestingly, axon orientation was also identified as a key pathway in our study. Classical neural guidance molecules have been implicated as regulators of vascular remodeling and vessel navigation, such as netrins, semaphorins, and ephrins [55,56]. In particular, secreted class 3 semaphorins (SEMA3) were confirmed as effective normalizing agents of cancer vasculature [57].

The activation of Ras triggered by GTP binding ultimately leads to mitosis in fibroblasts and epithelial cells via the RAF/MEK/ERK cascade and the PI3K/AKT downstream pathway. Oncogenic Ras could directly induce the upregulation of EMT-activated transcription factors, and promote cell invasion and migration [58]. On the other hand, the phosphorylation of the TGF*β* receptor provided docking sites for SH2 domain-containing proteins such as PI3K, GRB2, and SOS, thus linking it to the PI3K and Ras pathways [59,60]. The cooperation of the Ras and TGF*β*-non-Smad signaling pathways can induce the EMT process and tissue fibrosis in tumors. Furthermore, Ras-mediated ERK-MAPK activation promoted angiogenic homeostasis by increasing the levels of pro-angiogenic factors in endothelial cells [61].

Rap1, a member of the Ras superfamily, has been reported to participate in the regulation of cell adhesion, polarity, and cellular interactions during fetal development [62]. Previous studies have shown that Rap1 affects cell adhesion and related EMT process by targeting two major factors, cadherin and integrin [63]. The downregulation of *miR-708* in ovarian cancer cells resulted in the suppression of Rap1b, thereby impairing integrin-mediated cellular junction formation, migration, and invasion [64]. Additionally, Rap1 could promote VEGFR2 activation in endothelial cells, and induce angiogenesis by regulating integrin *α*v*β*3 [65]. Therefore, the abnormal level of miR-708 and aberrant signaling of Rap1 might be vital factors affecting peritoneal structural changes or fibrosis in patients with ultrafiltration failure. Of note, Rap1 has a high sequence identity with Ras, and can competitively bind with Raf1 effectors to attenuate Ras-mediated ERK activation [66]. With these reports, together with our results, it can be speculated that ubiquitin-mediated proteolysis, axon orientation, the Rap1 signaling pathway, and the Ras signaling pathway may be associated with the peritoneal structural changes and ultrafiltration failure in patients with ESRD.

However, this study has certain limitations. First, our conclusions need to be verified in another study with a larger sample size, and more experiments need to be conducted to validate the target genes of these DE-miRNAs through the dual-luciferase reporter gene assay of functional assays. The specific roles of miR-708-5p, miR-1273c-p-3, miR-1277-5p, miR-132-3p, miR-296-3p, and miR-125a-5p in ultrafiltration failure should also be explored, and more evidence is needed to support the identification of these DE-miRNAs as biomarkers used for the diagnosis of ultrafiltration failure. Additionally, the biological functions and key pathways of candidate miRNAs in peritoneal fibrosis will be investigated in subsequent studies and further experimental verifications also need to be performed.

## 5. Conclusions

In this study, exosomes were isolated from the PDE of failed and successful ultrafiltration and sent for small RNA sequencing. According to the sequencing results, 70 DE-miRNAs involved in the ultrafiltration failure were identified, including 41 upregulated and 29 downregulated ones. Besides, miR-125a-5p, miR-1273c-pc-3, miR-1277-5p, miR-132-3p, miR-296-3p, and miR-708-5p were found to be associated with peritoneal fibrosis by regulating angiogenesis and EMT. Functional analyses showed that ubiquitin-mediated proteolysis, axon orientation, the Rap1 signaling pathway, and the Ras signaling pathway may be associated with the peritoneal structural changes and ultrafiltration process. Our findings implied that these DE-miRNAs could be used as potential biomarkers of ultrafiltration failure, which would help us to discover novel therapeutic targets/pathways for ultrafiltration failure in patients with ESRD.

## Figures and Tables

**Table 1:** Clinical characteristics of patients.

Group		Ultrafiltration failure (*n* = 3)			Control (*n* = 3)		
		1	2	3	1	2	3
Age(years)		57	46	53	47	42	33
Sex		F	F	F	F	F	M
Duration of PD (month)		87	165	72	7	4	3
Infection times during PD		3	4	3	0	0	0
UF 360 min (mL)		430	230	390	670	430	720
Residual urine volume (mL)		0	100	185	180	600	150
Residual renal function (ml/min)		0	0.68	0.9	2.6	6.1	5.3
CRP (mg/L)		8.9	4.6	2.7	1.2	0.5	2.1
Hgb level (mg/dL)		91	85	93	102	91	84
Creatinine (*μ*mol/L)		886.5	1026.1	1128.6	720.9	633.2	823.9
Urea nitrogen (mg/dL)		22	25.7	23.5	20.4	15.8	25.2
PTH (pg/ml)		383.9	304.5	264.8	339.2	152.3	275.2
Serum calcium (mmol/L)		2.92	2	2.17	2.12	2.05	2.12
Serum phosphorus (mmol/L)		2.09	1.22	1.97	0.67	0.92	1.69
Albumin (g/L)		29.6	35.4	28.8	35.1	38.3	34.9
Total KT/V		1.94	2.17	2.14	2.55	2.9	2.21
D/Pcr (4 h)		0.92	0.97	1.01	0.69	0.59	0.60
D/Pna (1 h)		0.657	0.621	0.576	0.863	0.874	0.859
UF volume		30	−100	−150	450	500	600
MTAC (ml/mim)	BUN	24.51	26.63	28.98	15.21	15.88	16.80
	Scr	16.68	17.65	19.65	8.97	9.92	9.32
	UA	14.71	15.85	16.48	7.89	8.72	8.12

Note. UF: ultrafiltration failure; PTH: parathyroid hormone; D/Pcr: dialysate/serum creatinine concentration ratio; D/Pna: a sodium sieving ratio at 1 h; MTAC: material transport area coefficient of creatinine; BUN: blood urea nitrogen; Scr: serum creatinine; UA: blood uric acid.

**Table 2:** The sequences of all primers.

Primers	Sequence (5′-3′)
hsa-miR-1273c-p3-JH	GTCGTATCCAGTGCAGGGTCCGAGGTATTCGCACTGGATACGACTTGGGC
hsa-miR-1273c-p3-F	CAGAGTCTCGTTCTGTT
hsa-miR-708-5p-JH	GTCGTATCCAGTGCAGGGTCCGAGGTATTCGCACTGGATACGACCCCAGC
hsa-miR-708-5p-F	GCGCAAGGAGCTTACAATCTA
hsa-miR-155-5p-JH	GTCGTATCCAGTGCAGGGTCCGAGGTATTCGCACTGGATACGACAACCCC
hsa-miR-155-5p-F	GCGCTTAATGCTAATCGTGATA
hsa-miR-125a-5p-JH	GTCGTATCCAGTGCAGGGTCCGAGGTATTCGCACTGGATACGACTCACAG
hsa-miR-125a-5p-F	GCGCTCCCTGAGACCCTTTAAC
hsa-miR-25-5p-JH	GTCGTATCCAGTGCAGGGTCCGAGGTATTCGCACTGGATACGACCAATTG
hsa-miR-25-5p-F	GCAGGCGGAGACTTGGG
hsa-miR-132-3p-JH	GTCGTATCCAGTGCAGGGTCCGAGGTATTCGCACTGGATACGACGACCAT
hsa-miR-132-3p-F	GCGCTAACAGTCTACAGCC
hsa-miR-1277-5p-JH	GTCGTATCCAGTGCAGGGTCCGAGGTATTCGCACTGGATACGACATACGT
hsa-miR-1277-5p-F	GCCGCGGAAATATATATATATATGT
hsa-miR-296-3p-JH	GTCGTATCCAGTGCAGGGTCCGAGGTATTCGCACTGGATACGACGGAGAG
hsa-miR-296-3p-F	GCGAGGGTTGGGTGGAGG
Universal primer	GTGCAGGGTCCGAGGT
JH-cel-miR-39-RT	GTCGTATCCAGTGCAGGGTCCGAGGTATTCGCACTGGATACGACCAAGCT
cel-miR-39-F	GGCCTCACCGGGTGTAAATCAG

## Data Availability

The data that support the findings of this study are openly available in the Gene Expression Omnibus database at https://www.ncbi.nlm.nih.gov/geo/query/acc.cgi?acc=GSE182736, and the reference number is GSE182736.

## Abbreviations

miRNA: MicroRNAs
ESRD: End-stage renal disease
PDE: Peritoneal dialysis effluent
EMT: Epithelial–mesenchymal transition
PET: Peritoneal balance test
PMC: Peritoneal mesenchymal cells
GO: Gene Ontology
KEGG: Kyoto Encyclopedia of Genes and Genomes.
